# A qualitative study of health‐care experiences and challenges faced by ageing homebound adults

**DOI:** 10.1111/hex.13072

**Published:** 2020-05-31

**Authors:** Joyce M. Cheng, George P. Batten, Thomas Cornwell, Nengliang Yao

**Affiliations:** ^1^ University of Virginia College of Arts and Sciences Charlottesville VA United States; ^2^ Shandong University School of Health Care Management (NHC Key Laboratory of Health Economics and Policy Research) Jinan China; ^3^ University of Virginia Cancer Center Charlottesville VA United States; ^4^ Home Centered Care Institute Schaumburg IL United States; ^5^ University of Virginia School of Medicine Charlottesville VA United States

**Keywords:** health care, home‐based primary care, homebound, mobility limitations, older adults, qualitative interviews

## Abstract

**Background:**

The ageing of the global population is associated with an increasing prevalence of chronic diseases and functional impairments, resulting in a greater proportion of homebound individuals.

**Objective:**

To examine the health‐care experiences of older homebound adults who have not previously received home‐based primary care (HBPC). To explore their impressions of this method of care.

**Design:**

Cross‐sectional qualitative study using semi‐structured interviews.

**Setting and Participants:**

18 older homebound individuals in Central Virginia.

**Results:**

Our findings revealed that homebound individuals faced significant health challenges, including pain resulting from various comorbidities. They felt that their mobility was restricted by their physical conditions and transportation challenges. These were major barriers to social outings and health‐care access. Participants left their homes infrequently and typically with assistance. Regarding office‐based care, participants were concerned about long wait times and making timely appointments. Some thought that HBPC would be convenient and could result in better quality care; however, others believed that the structure of the health‐care system and its focus on efficiency would not permit routine HBPC.

**Discussion and Conclusions:**

Older homebound adults in this study faced high burdens of disease, a lack of mobility and difficulty accessing quality health care. Our observations may help researchers and clinicians better understand the health‐care experiences and personal opinions of older homebound individuals, informing the development of effective and empathetic home‐based care. Participant responses illuminated a need for education about HBPC. We must improve health‐care delivery and develop comprehensive, patient‐centered HBPC to meet the needs of homebound individuals.

## 1 INTRODUCTION

The proportion of older individuals in the United States is rapidly growing. The US Census projects that by 2050, more than 20% of the total US population will consist of older adults, compared with 13% in 2010.^1^ This ageing of the population is associated with an increasing prevalence of chronic illnesses and functional impairments, resulting in a greater proportion of homebound individuals, in relation to the US population as a whole.^2^ In 2011, 5.6% of people aged 65 and older (approximately 2 million people) were considered homebound.^3^ Homebound status is defined as never or rarely leaving the home in the last month, while semi‐homebound status is defined as needing assistance or having difficulty leaving the home.^3^ Homebound and semi‐homebound adults experience a range of illnesses that prevent them from easily leaving their homes and accessing hospitals, office‐based medical care, and social interactions.^2^ They experience metabolic, cardiovascular, cerebrovascular, and musculoskeletal diseases, as well as cognitive impairment and depression, at higher rates than the rest of the older population.^2^, ^4^ Loneliness, social isolation, and decreased life satisfaction associated with homebound status can also have adverse impacts on both mental and physical health conditions.^5^, ^6^

The health‐care needs faced by homebound older adults are associated with high costs to the US health‐care system.^3^, ^4^, ^7^ Homebound individuals’ difficulty accessing traditional office‐based primary care is associated with an increased number of emergency department visits and hospitalizations.^8^, ^9^ Homebound patients are estimated to account for around half of the costliest 5% of patients, due to their comorbidities, functional impairment, frailty, and social stressors.^10^, ^11^, ^12^ The top 5% of health‐care spenders were thought to be responsible for approximately 60% of all health‐care costs in 2011.^11^ The top 10% most costly beneficiaries of Medicare spending, which likely encompasses the majority of older homebound patients, incur Medicare payments 6.5 times the fee‐for‐service (FFS) average.^10^, ^13^, ^14^ These costs can be mitigated through home‐based primary care (HBPC), which provides comprehensive, interdisciplinary, and longitudinal care at home for individuals with chronic, complex conditions.^7^, ^9^, ^15^, ^16^, ^17^, ^18^ It is important to distinguish HBPC from Medicare home health‐care services, although both are often referred to as home‐based medical care, as Medicare home health care tends to be temporary or intermittent.^7^, ^9^, ^15^, ^17^, ^18^ HBPC produces high patient satisfaction rates, lower hospitalization durations, lower readmissions, decreased emergency department visits, and lower Medicare costs with similar survival outcomes.^3^, ^7^, ^9^, ^16^, ^17^, ^18^, ^19^, ^20^, ^21^, ^22^ There is currently a shortage of HBPC providers compared to the number of individuals in need.^7^, ^9^, ^16^

There is a lack of qualitative research designed to investigate the health‐care experiences and needs of homebound people and their perceptions of HBPC, specifically in the United States.^19^, ^23^ The objective of our study was to integrate patient narratives into this field, through interviews examining ageing homebound individuals’ perceptions of their interactions with the health‐care system. We intended to understand how becoming homebound affects an individual's ability to access health care. We were primarily interested in examining the opinions and attitudes of homebound adults (who were not receiving HBPC) on the concept of HBPC, and learning whether they would be open to this system of medical care. An advantage of our study was that it involved a relatively high proportion of rural and African American participants, as these populations tend to be underrepresented in health‐related research.^24^

## 2 METHODS

### 2.1 Study design and population

Participants were recruited from the city of Charlottesville, Virginia, and the surrounding Albemarle, Louisa, Buckingham, Fluvanna and Orange counties. Eligibility criteria for the study included homebound status, age 50 or older, fluency in English and no apparent cognitive impairment. Participants were recruited through convenience sampling from clinical programmes, community programmes that serve homebound patients, and through a snowball sampling method. Clinical programmes included University Physicians at the Jefferson Area Board for Aging (JABA), University Medical Associates and University of Virginia (UVA) Continuum Home Health, which serves both homebound and semi‐homebound individuals. University Physicians at JABA provides primary care to patients aged 65 and older in an office setting; University Medical Associates is also a primary care office. UVA Continuum Home Health provides home health services to patients of all ages who are disabled, chronically or terminally ill, or recovering from an acute illness. Community programmes included JABA Charlottesville, Mom's Meals and Meals on Wheels of Charlottesville/Albemarle. JABA is a 501(c)(3) non‐profit that serves older adults, individuals with disabilities and caregivers in Central Virginia. Mom's Meals and Meals on Wheels are programmes that deliver meals to individuals who may have decreased mobility; Mom's Meals is a for‐profit company under PurFoods LLC, whereas Meals on Wheels is a 501(c)(3) non‐profit. Snowball sampling is a recruitment technique by which participants suggest other potential participants for the study. Homebound individuals provided a name and phone number for potential participants; the researchers then contacted these individuals about the study. Homebound status for an individual was defined as being unable to leave one's home without assistance or being confined to the home for a majority of the week, encompassing both homebound and semi‐homebound common definitions. A loose definition was used due to a lack of consensus on the definition of homebound status and because definitions based on eligibility for Medicare services may not encompass the entire homebound population.^2^, ^25^, ^26^ To investigate homebound status, participants were asked about how often they leave home, who accompanies them, and the duration of their outings. The University of Virginia Institutional Review Board for Health Sciences Research (IRB‐HSR) provided approval for this study (IRB‐HSR #20909).

### 2.2 Data collection and analysis

During summer 2018, the primary author contacted health‐care providers and organizations in the Charlottesville community involved in homebound patient care, which advertised the study and distributed information about the study to potential participants. Interested older adults who received this information then contacted the researchers, who described the study and asked whether the individuals were willing to participate. Homebound status was verified over the phone, seeing as the eligibility criteria for the programmes from which participants were recruited were diverse and not limited to only homebound individuals. Willing participants reviewed and signed an IRB‐approved consent form and Health Insurance Portability and Accountability Act (HIPAA) authorization form.

A total of 18 interviews lasting an average of 50 minutes (range 26 to 96 minutes) were conducted at the homes of participants. A qualitative descriptive design was adopted, which used semi‐structured, guided interviews to create a detailed, in‐depth summary of the intricacies of homebound life.^27^, ^28^ Participants were asked about their social demographics, and personal perspectives regarding their health‐care challenges and physical, mental, and social situation. Using this method, topical questions posed to participants led to descriptive answers about their lived experiences, while still giving participants the flexibility to provide information not necessarily probed for by the study team. This approach was most appropriate, as it provided a summary of the complexities of homebound life from nuanced perspectives, leading to a greater understanding of the individual needs and specific dynamics among these particular ageing homebound participants.^29^

Interviews were audio‐recorded and transcribed verbatim. To ensure participants’ privacy, pseudonyms were assigned to individual participants, identifying information was removed, and files were stored on secured devices. Descriptive statistics were used to describe characteristics of the participants. The interview data were analysed using a thematic analysis approach via NVivo 12 software (QSR International) for qualitative coding. An a priori topical coding scheme was developed based on the interview guide and grounded in patient work system theory.^30^, ^31^ This model represents the self‐care performed by homebound participants as health‐related work, the processes of which affect health status but are also impacted by an individual's attributes, performed tasks, physical environment and social environment.^30^, ^31^ This model provided guidance for developing an initial coding structure around the unique characteristics of homebound health care.^30^, ^31^ Open coding was then used to capture emerging concepts.^32^ Inductive and deductive coding schemes were refined using an iterative process of constant comparison, based on an increasing understanding of the study data.^33^ New themes were identified and created until data saturation was reached and no new coding categories emerged; participant recruitment was then closed. To establish inter‐coder reliability (ie a consistent, unbiased application of thematic coding), two researchers independently coded a portion of transcribed interviews. They cross‐examined their individual coding—coding schemes were compared at both the beginning and conclusion of data analysis. If there were any inconsistencies or differing interpretations of the coding outcomes, a third member of the research team was available to review the codes and assure internal validity of the coding schematics. After these iterations, the final coding scheme was then applied to the interview data. The data that support the findings of this study are available on request from the corresponding author. The data are not publicly available due to privacy or ethical restrictions.

## 3 RESULTS

### 3.1 Demographic information

A majority of the 18 participants were female and the participants’ ages ranged from 52 to 91 years, with the average age being 68.7 years (Table 1). The sample was almost evenly split between Caucasian and African American participants. Most participants lived in Charlottesville and most lived alone. The highest education levels of the participants varied, ranging from some grade school to a college degree. These demographics are somewhat reflective of an epidemiologic study of the US homebound population by Ornstein et al using data from the National Health and Aging Trends Study, in that homebound individuals tended to be older, female, and non‐Caucasian, and have less education.^3^

**TABLE 1:** Participant characteristics

Characteristic	Number of participants (n = 18)	Percent (%)
Sex		
Male	5	27.8
Female	13	72.2
Age (years old)		
50‐59	3	16.7
60‐69	9	50.0
70‐79	3	16.7
80‐89	1	5.6
90‐99	2	11.1
Race		
Caucasian	8	44.4
African American	9	50.0
Asian	1	5.6
Area of residence		
Charlottesville	12	66.7
Surrounding counties	6	33.3
Living status		
Alone	13	72.2
With others	5	27.8
Highest education level		
High school diploma or less	9	50.0
Some college	6	33.3
Associate or bachelor's degree	3	16.7

### 3.2 Thematic results

Three major thematic clusters relevant to participants’ health‐care experiences as homebound individuals emerged during the interviews: (1) health conditions, (2) mobility limitations and (3) health‐care experiences. While other interesting themes did emerge, such as social isolation and spirituality, these three categories were articulated by the majority of the respondents and given the greatest emphasis in this analysis, due to our research focus on participant interactions with the health‐care system. In the description of the thematic results below, refer to Tables 2, 3, 4.

**TABLE 2:** Health concerns faced by homebound participants

Concerns	Quotes
Physical impairment	**1:** ‘It's nice to have to look and see you have two legs, but they have no strength’. **2:** ‘I’m a quadriplegic… I usually explain myself as being a big mouth, a big brain trapped in a nonfunctional body’.
Pain	**3:** ‘It hurts. I mean, like, at night, sometimes, it's like my feet are in a yellowjacket's nest. It's unreal. And there's nothing that can help’. **4:** ‘I feel like I'm being burned from inside by a thousand needles’. **5:** ‘Feels like somebody stabbed my leg and my foot feels like it's on fire’.
Depression	**6:** ‘Sometime I get to cry and get depressed’. **7:** ‘I get really stressed, and I really get depressed. I get lonely, I get bored’. **8:** ‘I don't know why I'm depressed, because, I don't know’.

**TABLE 3:** Mobility limitations faced by homebound participants

Limitations	Quotes
General mobility challenges	**9:** ‘I can't get around like I used to… I used to walk a whole lot. I can't do it’. **10:** ‘It makes me sad that I couldn't motivate [ambulate], like I used to, you know, and just go hop in the car and go shopping or go where I want to go’. **11:** ‘Everything has changed because I can't get out and go places’.
Limitations on outing destinations	**12:** ‘If I'm going somewhere it's because I'm going to go to the grocery store or I'm going to go to the hospital’. **13:** ‘I go and see people when I go out, I see family, friends, and things when I’m out’.
Outing enjoyment	**14:** ‘Most of the time I enjoy them [outings] a lot because it gets me out of the house’. **15:** ‘I like Walmart. Where I can go and get my own food. And, and get what I want to eat’.
Outing difficulty	**16:** ‘I do it [going out] ‘cause I have to. It's not really fun to go out with the walker’. **17:** ‘I don't want to go. Have to push myself to go. I'd rather be at home on the sofa’. **18:** ‘I go to Health South, and then there's nobody there that helps you out of the car’. **19:** ‘I, it's very difficult for me to get down to the bus stop by myself because the sidewalks are not made in Charlottesville to accompany a wheelchair. With the walker, one time I hit a lip on the cement and I fell and broke my leg’.
Transportation limitations	**20:** ‘I got to get on a bus or Jaunt or something, and sometimes, somebody would come and take me but it's kind of hard’. **21:** ‘My daughter takes me, people… friends in the neighborhood, my kids’. **22:** ‘I feel like I'm a burden to them. Hey, I don't want to be like this. I don't want to sit around and be hopeless—not hopeless, but helpless all the time’.

**TABLE 4:** Health‐care experiences of homebound participants

Experiences	Quotes
Health‐care access	**23:** ‘You gotta wait months and then, I, unless I write it down ahead of time, my memory isn't all that good… sometimes, I miss an appointment, because it's four months down the road’. **24:** ‘I don't, just don't have the money to do it… so I have to go without something I need, and I know, my teeth really impacts my health care… when you get older, you need that dental care’. **25:** ‘There's really no follow up on an emotional level… they [doctors] wanted to make sure I was mending, but there's no one to find out how you're mending psychologically’.
Health‐care efficiency	**26:** ‘It's their schedule. I think they have maybe too many patients sometimes’. **27:** ‘I'm gonna sit in the damn office for 8 hours? Diabetic? That's… that's bad practice’. **28:** ‘The main thing is, with some doctors, it's all about turning the dollar. Let's get this guy in, and get out. You cannot know a person in 15 minutes’.
Emergency care	**29:** ‘I think they [doctors] are overworked, and there's not enough time to really treat patients in the emergency room. So, I tell people if at all possible, avoid the emergency room’. **30:** ‘They have a lot of pressure, so they don't have time to concentrate on… when you got a lot of patients coming in with different ailments, you know, they're not going to just concentrate on me’. **31:** ‘I go to the emergency room when I'm having an issue and they just do the minimum that they have to do to get rid of me, cause then they send me bills for thousands of dollars that I can't pay’.
Provider characteristics	**32:** ‘He listens to me… he cares about my care… he explains stuff to me’. **33:** ‘I would say the most important thing I could impart would be for them to listen’. **34:** ‘You have to care about the patient, you have to be willing to take care of them and not only be there for the money’. **35:** ‘Having a bedside manner, knowing how to talk to a patient’.
Patient self‐knowledge	**36:** ‘I think doctors, first of all, need to listen to their patients. Patients are pretty much the experts on their particular body’. **37:** ‘A person knows what their body is feeling like, not the doctor’. **38:** ‘They don't look into… they don't listen to the patient. They don't feel my pain. I sometimes wish I could radiate my pain to the doctors that I'm seeing, so they can feel what it's like’.
Patient‐provider relationship	**39:** ‘I would much rather have a doctor that I have confidence in, and stick with him. So over the years you have a rapport and then a sort of relationship’. **40:** ‘You can't care for somebody unless you know who they are’. **41:** ‘Then the patient feel more comfortable of telling them everything, instead of just, you know, feel like I'm just gonna check you out, and they just, you know, this just another number, just to get a dollar’.
Support for home‐based care	**42:** ‘It's so much easier to give better care, to get better care when you're in a home situation. You're more comfortable around, in your own setting, around your own things, around your own family’. **43:** ‘I think that [HBPC] would be very good. You don't have to worry about the struggle of getting out of there and then some time if you ride and like the Medicaid van and stuff like that, you gotta wait for hours for them to come back to pick you up’. **44:** ‘They ask you questions when you're in the hospital… people can say things and it's not true, but if a doctor goes to the house, he can see’.
Opposition to home‐based care	**45:** ‘I got so used to going to them [doctors]. I’m set in my own ways like that’. **46:** ‘Sometimes I think it's not better because they have to waste their time driving somewhere. I can't see that that's feasible’. **47:** ‘It [HBPC] would really make me not want to go out at all, so, by going out to them [doctors], it is getting me out of the house some’.

#### 3.2.1 Health conditions

The homebound individuals interviewed experienced various health problems. Common physiological illnesses encompassed arthritis, diabetes, stroke, bone fractures, cardiovascular disease, heart failure, high cholesterol and urinary tract infection. Pain was mentioned by 12 participants and 16 participants considered themselves physically disabled in some manner (quotes 1‐5). Psychological conditions included depression, anxiety, schizophrenia, attention deficit disorder and post‐traumatic stress disorder. Depression was a prevalent influence on homebound individuals’ mental health; 10 participants mentioned feeling depressed, although not all of them have been clinically diagnosed (quotes 6‐8). For a majority of the older adults interviewed, the development of homebound status was due to a physical impairment resulting from medical conditions such as arthritis, bone fractures and stroke. Almost all participants experienced at least two major medical conditions.

#### 3.2.2 Mobility limitations

A majority of participants were only able to leave their home 1‐3 times per week, typically only for grocery shopping or to attend a doctor's appointment (quotes 9‐12). Mobility restrictions were due to physical disabilities, lack of transportation and financial constraints. Most participants were either driven by family or friends or used public transportation services, such as a regional bus service for ADA‐certified riders providing curb‐to‐curb or door‐to‐door transport, depending on location, by appointment (quotes 20‐21). Public transportation posed challenges for study participants who were wheelchair or walker‐bound, although some individuals said transportation employees helped with access onto the vehicle (quote 20). However, getting to a public bus stop and getting from drop‐off locations to office and hospital entrances remained additional obstacles (quote 19). Participants expressed that depending on other people to get around made them feel like a burden and prevented them from engaging in community social activities (quote 22). Transportation limitations posed an even greater difficulty to rural participants, who needed to travel farther to access office‐based health care.

Participants both enjoyed their outings and found them challenging. Many participants wished they could still go out socially or engage in activities they used to enjoy, and variations of the phrase ‘I wish I could do the things I used to do’ came up in a majority of the interviews (quote 10, quotes 14‐15). Even grocery shopping allowed participants a sense of autonomy because they could browse the store and make independent decisions about what to purchase (quote 15). However, some participants did respond that although they could not leave their homes often, they would rather stay at home regardless, as their outings caused them physical discomfort, pain, anxiety or fear (quotes 16‐19).

#### 3.2.3 Health‐care experiences

Participants were asked a series of questions pertaining to their personal health‐care experiences and their opinions on the concept of home‐based primary care. A common complaint among participants concerned the efficiency of the health‐care system, given that homebound older adults often faced long wait times in medical settings (quotes 26‐28) and encountered delays when trying to schedule new office‐based appointments (quote 23). Participants often felt rushed or undertreated during urgent care or emergency room visits (quotes 28‐31). From a patient perspective, rushed appointments gave the impression that the health‐care system prioritizes efficiency and profit over the needs of patients (quote 28, quote 31). Multiple participants referenced themselves as being experts of their own bodies and felt like some doctors formed preconceptions of their patients’ disease, failing to understand their pain (quotes 36‐38). One study participant raised a unique point regarding health‐care services for the older homebound population, saying that following a hip replacement operation, although doctors ensured she was mending physically, there was a lack of follow‐up on her mental health (quote 25). Three participants cited a lack of accessible, affordable dental care for homebound older adults, which compromised their oral health, as dental disease is a largely preventable burden (quote 24). The rural participants were especially affected by a lack of dental services.

When asked which characteristics and behaviours they believed an effective care provider should display, participants referenced bedside manner and interpersonal communication skills (quotes 32‐35). Most participants interviewed had encountered some form of Medicare home health care, such as physical therapy or personal caregivers, but none routinely received home‐based primary care. When questioned about their opinion on HBPC, participants reported mixed opinions. Some participants believed that the structure of the health‐care system and its focus on efficiency would not permit routine HBPC (quote 46). A few participants said they were used to going out to see their health‐care providers, and it got them out of the house (quote 45, quote 47). Many participants agreed that HBPC would be convenient and could result in better health care as practitioners may better understand one's living environment (quotes 42‐44); however, other participants were uncomfortable with the prospect of having clinicians in their home. In addition, some individuals wanted to continue seeing their trusted long‐time personal physician, in office (quote 39, quote 45).

## 4 DISCUSSION

Participants shared a collection of compelling narratives regarding their experiences as homebound older adults in relation to the health‐care system. Although responses to more sensitive personal topics were likely influenced by participants’ unique experiences and personality, common opinions that fell within the thematic clusters of health conditions, mobility limitations and health‐care experiences still arose often. By speaking about their health conditions, participants helped characterize the heavy medical burden faced by older homebound individuals. Medical conditions that resulted in functional limitations and homebound status seemed to limit access to office‐based care. This challenge was exacerbated by public transportation inaccessibility. In describing their interactions with the health‐care system, participants indicated issues with office‐based care, desirable health‐care provider characteristics and varied opinions of home‐based primary care. These themes were supported by existing qualitative research and are important considerations for health‐care providers and policymakers involved in the care of homebound older adults.^23^, ^34^

In accordance with published research on the homebound population, the participants interviewed faced significant physical and mental health challenges.^2^, ^4^, ^34^ Physically, pain and disability posed the largest burden on study participants’ functionality. Participants often conveyed that they felt like their doctors did not understand their level of pain or treat it effectively. Perhaps, these reports illuminate a lack of attention towards the pain of older people, which tends to be undertreated.^35^, ^36^ Although it is critical not to over‐treat pain, chronic pain should be adequately addressed because of its association with negative emotions, depression and decreased physical functioning.^37^, ^38^, ^39^ A clinical review of pain management in older adults recommends an integrated approach that uses a combination of pharmacologic and rehabilitative methods and cultivates a strong therapeutic alliance between the patient and clinician.^39^ These findings are consistent with participants’ opinions from our study; participants frequently reported that they appreciated physical therapy and felt that it improved their functional abilities. Participants also favoured health‐care providers who listened to the patient's concerns and opinions, and collaborated with the patient to formulate a treatment plan.^23^

Homebound individuals wanted to be more mobile and independent, and felt restricted by their physical conditions and transportation challenges.^23^, ^34^ Compared to participants who lived in Charlottesville, rural participants experienced a higher travel burden.^40^, ^41^ In emergency situations, study participants were often forced to call an ambulance for assistance, which involved a high, often unaffordable cost. Most patients enjoyed the independence and autonomy associated with the ability to go out. Difficulties with going out and accessing office‐based health care may be mitigated through increased provision of services such as insurance‐provided non‐emergency medical transportation (NEMT), increasing public infrastructure accessibility and increasing education about available transportation assistance services.^42^, ^43^ For individuals unable or unwilling to leave their homes, HBPC could play a major role in improving patients’ health through avoiding the cost, burden and inconvenience associated with transportation challenges, and through increasing access to health care.^20^, ^21^, ^22^

Among the most prevalent concerns regarding office‐based care were difficulty with making timely appointments and long wait times. Participants highly valued health‐care providers who listened closely to patients and displayed genuine care and concern. Coordinated HBPC where patients’ goals and preferences are clear, patients are provided community resources, and collaboration exists between health‐care providers, social workers and medical equipment agencies has the potential to circumvent office‐based care issues.^15^, ^16^, ^17^, ^18^, ^26^, ^34^, ^44^ However, study participants held diverse opinions about home‐based primary care. Some participants believed that HBPC would be convenient because individuals are more comfortable in their own homes and they could avoid the time, expenses and physical difficulty of getting to office‐based primary care.^15^, ^26^, ^44^ HBPC also has the potential to lead to better patient‐provider understanding and overall medical care, because care providers could observe patients’ living situation and offer suggestions to improve patients’ health and safety.^34^, ^45^ Nevertheless, some participants hesitated at the idea of receiving HBPC, stating that doctor's appointments were among the only reasons they left their homes, which they wanted to do to preserve their autonomy, despite difficulties. Participants who had never encountered HBPC suggested they would prefer continuing to go to the office of a trusted long‐term physician, out of habit. Others associated HBPC with strangers coming into their private homes and felt like this could be an uncomfortable invasion of privacy. Lastly, a number of participants were concerned that HBPC would make the health‐care system less efficient, due to time and resources required for care providers to travel to patients’ homes.

As homebound older adults possess widely varying opinions towards HBPC, one's individual needs and preferences should be considered when deciding whether to implement HBPC for the particular individual. Opposition and confusion regarding HBPC may be influenced by a lack of exposure, experience and education about this system of medical care, which has only recently experienced a resurgence.^7^, ^26^ The responses given by participants in our study resembled this quote presented by Leff and Burton, from a patient when told they could be seen in their home: ‘I didn't know anyone did that anymore’.^46^ A few study participants spoke about having physicians come to their homes when they were children, nearly 60 years ago. Some participants were concerned about the effect of HBPC on health‐care system efficiency; however, technological advances, new payment models and varied HBPC models may actually improve efficiency and cost‐effectiveness.^7^, ^16^, ^20^, ^21^, ^46^ Furthermore, HBPC provides an opportunity for strong, long‐term patient‐provider relationships, just as office‐based visits do, through regular home calls conducted by the patient's interdisciplinary care team.^7^, ^46^ Participants should be made aware of HBPC as an option, perhaps through receiving information from their current clinicians and insurance companies about available programmes near them. They could also be presented with information upon visits to urgent care centres or the emergency department. Being aware of available options will increase individuals’ health literacy, giving them autonomy in making the health‐care choices best suited to their needs. While office‐based care may still be preferable for individuals who maintain the ability to leave their home without significant difficulty or pain, as well as those who shared privacy concerns, HBPC may be a better option for those facing more restrictive functional disabilities. Furthermore, for individuals challenged by considerable ADL deficiencies, HBPC may lessen the incidence of ambulance trips to the emergency department.^9^, ^19^, ^20^, ^21^, ^22^ The worry that receiving HBPC would cause homebound individuals to lose their incentive to leave their homes could be mitigated if homebound older adults were offered resources and accessible transportation to reach local senior centres, churches and other desired destinations. Individuals deserve to know about all viable health‐care options available to them. Aside from discussing HBPC, a few participants raised unique and interesting concerns regarding a lack of attention towards mental health and dental care for homebound individuals that should be addressed in further research and health‐care practice.

### 4.1 Study limitations

While this research illuminates important perspectives of the homebound older population, it has various limitations. There are limitations to the recruitment method; participants learned about the study from common programmes they utilize, so they may have shared characteristics. For example, participants recruited through UVA Continuum Home Health may tend to have more positive conceptions of home‐based care, having received some form of in‐home care, whether it be physical therapy or home‐based social work services. Disadvantages of snowball sampling include lack of representativeness and sampling bias, as participants may refer potential participants with similar characteristics due to homophily. The recruitment method did not appear to exhibit any association with severity of participant health problems based on interview data; however, no formal analysis based on recruitment method was performed. Increased diversity of the participant population might have revealed additional relevant perspectives that are culturally significant or unique. Although the proportion of Asians and Latinx individuals in Central Virginia is low (around 3%‐6% based on estimates for various counties), additional data should be collected from these groups to obtain opinions that are more representative of these populations. The relatively large number of rural and African American participants in this study is a strength, as it illuminates important, often underrepresented viewpoints.

Due to the qualitative nature of our research, no causation nor statistically significant correlations can be drawn from the study's observations. Furthermore, variations in regional culture and preferences prohibit this qualitative investigation from being generalizable nationwide. Study participants mentioned financial difficulties in relation to transportation, hospital visits and office‐based care, but no empirical data on socio‐economic status were collected. In future studies, it would be valuable to collect this information and investigate correlations between particular participant characteristics and their opinions on health care. Additionally, future research involving a larger sample could explicitly compare the experiences of urban versus rural populations. Future research on this overarching topic should focus on investigating a specific thematic category highlighted by this study, using quantitative statistical methods and a larger, more diverse sample that is more representative of and generalizable to the population.

## 5 CONCLUSION

The key thematic categories of health conditions, mobility limitations and health‐care experiences characterized by this study provide a framework for further investigation into factors that affect the lives of homebound older adults. Homebound individuals in this study and in the literature faced a higher disease burden than the rest of the older population. They drew specific attention to the physical pain they commonly experienced and the manner in which it was addressed by their health‐care providers. Mobility restrictions resulting from physical impairments and lack of transportation limited individuals’ access to health care and social activities, and rural individuals were especially disadvantaged. Homebound older adults utilize health care at high rates, so it is imperative to consider their opinions on the quality and efficacy of health‐care services. These results highlight a lack of knowledge regarding home‐based primary care. This research may help researchers, clinicians and policymakers better understand the health‐care experiences of older homebound adults in the United States. This study should inform the development of comprehensive patient‐centered home‐based care, as well as new approaches for overcoming identified health‐care challenges.

## CONFLICT OF INTEREST

The authors declare that there is no conflict of interest.

## AUTHOR CONTRIBUTIONS

Yao and Cheng conceived and designed the study. Cheng acquired the data and drafted the manuscript. All authors analysed and interpreted the data and critically revised the manuscript.

## Data Availability

The data that support the findings of this study are available on request from the corresponding author. The data are not publicly available due to privacy or ethical restrictions.
